# Normal social seeking behavior, hypoactivity and reduced exploratory range in a mouse model of Angelman syndrome

**DOI:** 10.1186/1471-2156-12-7

**Published:** 2011-01-14

**Authors:** Melody Allensworth, Anand Saha, Lawrence T Reiter, Detlef H Heck

**Affiliations:** 1 Department of Anatomy and Neurobiology, University of Tennessee Health Science Center, Memphis, TN 38139, USA; 2 Department of Neurology, University of Tennessee Health Science Center, Memphis, TN 38139, USA; 3 Department of Pediatrics, University of Tennessee Health Science Center, Memphis, TN 38139, USA

## Abstract

**Background:**

Angelman syndrome (AS) is a neurogenetic disorder characterized by severe developmental delay with mental retardation, a generally happy disposition, ataxia and characteristic behaviors such as inappropriate laughter, social-seeking behavior and hyperactivity. The majority of AS cases are due to loss of the maternal copy of the *UBE3A *gene. Maternal *Ube3a *deficiency (*Ube3a*^m-/p+^), as well as complete loss of *Ube3a *expression (*Ube3a*^m-/p-^), have been reproduced in the mouse model used here.

**Results:**

Here we asked if two characteristic AS phenotypes - social-seeking behavior and hyperactivity - are reproduced in the *Ube3a *deficient mouse model of AS. We quantified social-seeking behavior as time spent in close proximity to a stranger mouse and activity as total time spent moving during exploration, movement speed and total length of the exploratory path. Mice of all three genotypes (Ube3a^m+/p+^, *Ube3a*^m-/p+^, *Ube3a*^m-/p-^) were tested and found to spend the same amount of time in close proximity to the stranger, indicating that *Ube3a *deficiency in mice does not result in increased social seeking behavior or social dis-inhibition. Also, *Ube3a *deficient mice were hypoactive compared to their wild-type littermates as shown by significantly lower levels of activity, slower movement velocities, shorter exploratory paths and a reduced exploratory range.

**Conclusions:**

Although hyperactivity and social-seeking behavior are characteristic phenotypes of Angelman Syndrome in humans, the *Ube3a *deficient mouse model does not reproduce these phenotypes in comparison to their wild-type littermates. These phenotypic differences may be explained by differences in the size of the genetic defect as ~70% of AS patients have a deletion that includes several other genes surrounding the *UBE3A *locus.

## Background

Angelman syndrome (AS) is a neurogenetic disorder characterized by severe developmental delay with mental retardation, language delays, increased susceptibility to seizures and unique behavioral characteristics such as inappropriate laughter, social-seeking behavior and hyperactivity [1,2]. The prevalence of AS in the general population is between 1:10,000 and 1:40,000 [3]. Most cases of AS are caused by loss of function of the maternal copy of the *UBE3A *gene, although paternal uniparental disomy, imprinting center mutations and maternally inherited loss of function mutations in *UBE3A *can occur as well [4,5]. The *UBE3A *gene encodes an E3 ubiquitin protein ligase, which is involved in targeting proteins for mono-ubiquitination and subsequent cellular processes such as degradation or trafficking to other parts of the cell. In the brain *UBE3A *exhibits preferential maternal allele expression with highest levels of expression in the cerebellum and hippocampus [6]. Behavioral abnormalities of AS include inappropriate laughter, a generally happy disposition, impaired verbal skills, social dis-inhibition or social seeking behavior and hyperactivity [1-3]. Social seeking behavior is expressed as a dis-inhibited and unselective desire of AS individuals to seek attention from both caregivers and strangers.

Some studies have reported overlapping features between autism and AS especially when the maternally inherited deletions include not only the *UBE3A *gene, but also genes like *CYFIP1*, a gene coding for a protein that directly interacts with the Fragile X protein FMRP, that are proximal to the *UBE3A *locus [7,8]. However, social seeking behavior is uncharacteristic of autism and AS patients show a strong interest in social interaction from early infancy [9]. With their verbal communication impaired, these individuals typically communicate through facial expressions, gestures and body postures [7,10].

Jiang and colleagues were the first group to engineer a mouse model of AS via knock out of exon 2 of the mouse *Ube3a *gene [11]. The *Ube3a *deficient mouse model is often studied in the context of molecular studies that are aimed at finding the many roles of the *UBE3A *gene [6]. Only a few behavioral studies have been performed with *Ube3a *deficient mice [11-13] and none have addressed the social behaviors in the mouse model as compared to AS individuals. Moy *et al. *used a social interaction task similar to the one employed here to study social behavior in five mouse lines with genetic defects known to be related to Autism Spectrum Disorders in humans [14]. The behavioral abnormalities in these mice included a reduced social interest suggesting that the genes involved affected social behavior in both humans and mice.

The purpose of this study was to investigate social interest in a stranger mouse and exploratory activity in a mouse model of Angelman syndrome. We hypothesized that the human hyperactivity and social seeking phenotypes found in AS patients would be reproduced in *Ube3a *deficient mice.

## Results

All tests were performed with adult male mice 8 - 12 weeks of age that were either homozygous for the *Ube3a *null allele (*Ube3a*^*m-/p-*^, *n *= 11) or inherited the null allele through the maternal germline (*Ube3a*^*m-/p+*^, *n *= 25 except for measurements of area covered where *n *= 23). Wild-type littermates (*Ube3a*^*m+/p+*^, *n *= 36) of these animals were used as controls. The construction and breeding of the *Ube3a *deficient mice have been described by Jiang and colleagues [11].

**Figure 1:**
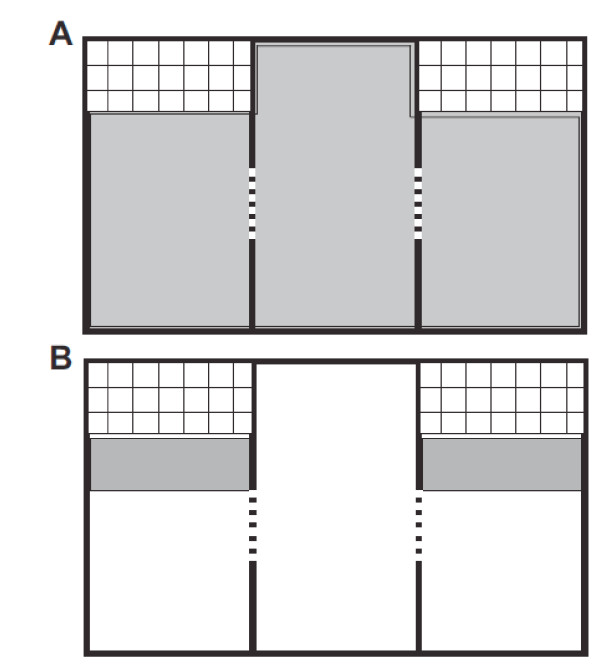
**Schematic drawing showing the outline of the arena with its three chambers and the two small cages and the areas used for movement path analysis**. The dividing walls had doors in the center (indicated by dashed lines) through which the mice could freely move between chambers. (A) The area shown in gray is the area within which the mouse's movements were tracked for the analysis of behavior across the whole arena. (B) For the analysis of social interest in the stranger mouse the analysis of the movement paths was limited to the two gray rectangular areas in front of the two cages.

The results section is divided into two segments: first, we report results from analyzing the entire length of the path, covering all of the arena (gray area in Figure 1A) under control conditions (with the two small cages empty) and under test conditions (with a stranger mouse present in one of the cages). Second, we describe results from analyzing the behavior directly in front of the two small cages, differentiating between the empty cage and the cage with the stranger mouse. Here, pathway analysis was limited to within the two small zones shown in gray in Figure 1B.

### Whole Arena Measurements

**Figure 2:**
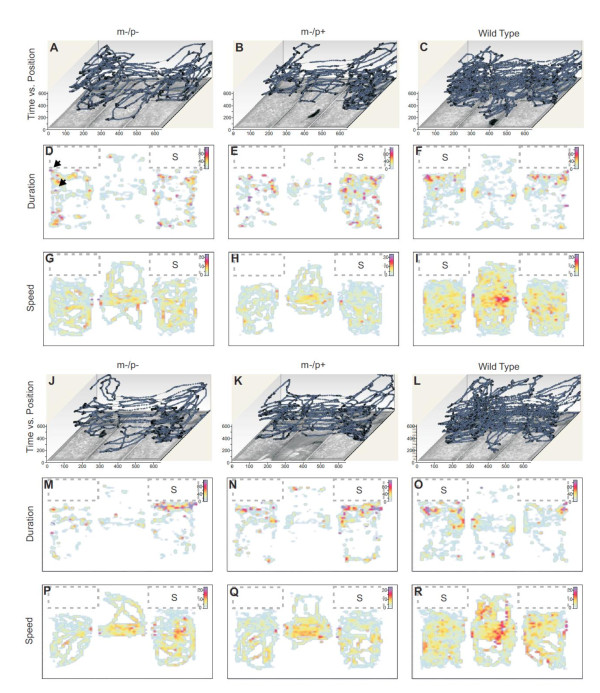
**Three-dimensional and color coded visualizations of different aspects of exploratory behavior**. Three aspects of exploratory behavior are shown under control conditions (A-I) and under test conditions, i.e. with a stranger mouse present in the cage marked with "S" (J-R). Each panel covers the entire arena. Data for *Ube3a*^m-/p-^, *Ube3a*^m-/p+ ^and wild-type mice are shown in the left, middle and right columns respectively. All behavioral variables visualized here for qualitative comparison were also compared quantitatively (see text for details). (A-C and J-L) "Time vs. Position" plots illustrate the position of the animal over time with time increasing along the z-axis. Movement paths recorded under control and test conditions are shown in (A-C) and (J-L) respectively. Duration (i.e. the time spent at any given site along the path) is represented in color-coded maps for control and test conditions in panels (D-F) and (M-O) respectively. Sites where mice lingered for ~60 seconds or more show up as red and purple spots (the two black arrows in (D) point at two examples of such sites). The speed with which mice moved along their exploratory path is shown in color-coded maps for control and test conditions in panels (G-I) and (P-R) respectively. The color scale for duration is time in seconds. The color scale for speed is in cm/sec.

Representative examples of exploratory behavior of *Ube3a *deficient and wild-type mice under control and test conditions (without and with a stranger mouse present, respectively) are visualized for qualitative comparison in Figure 2 as movement path vs. time plots (Figure 2A-C, J-L), and color maps for duration (Figure 2D-F, M-O) and movement speed (Figure 2G-I, P-R). Quantitative comparisons are shown in subsequent figures.

**Figure 3:**
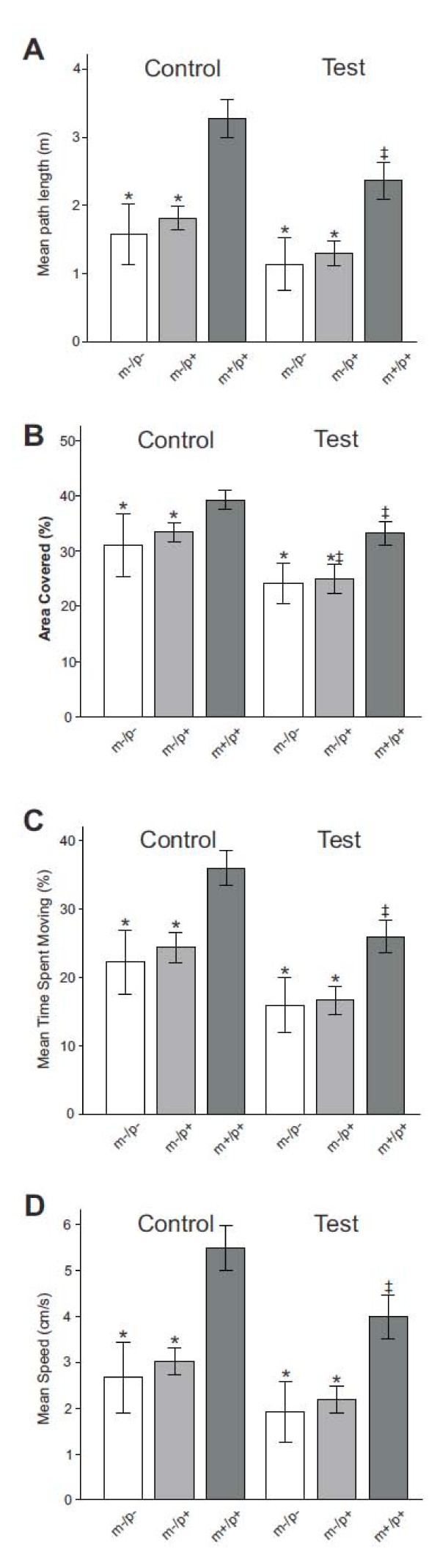
**Various measures of exploratory activity are significantly lower in *Ube3a *deficient mice compared to wild-type littermates**. Each bar graph shows results from control trials on the left and from test trials (with stranger present) on the right. Bar colors correspond to genotypes (*Ube3a*^m-/p- ^= white bar, *Ube3a*^m-/p+ ^= light gray, wild-type = dark gray). (A) The total length of the exploratory path was significantly shorter for *Ube3a *deficient compared to wild-type mice. (B) The area covered by the exploratory path was significantly smaller for *Ube3a *deficient compared to wild-type mice. For all three genotypes the area covered was larger during the control trial (left) than during the test trial. (C) *Ube3a *deficient mice spent significantly less time moving than wild-type mice during both control and test conditions. (D) The average movement speed was significantly lower for *Ube3a *deficient compared to wild-type mice. Symbols: * indicates a significant difference between the *Ube3a *deficient and the wild-type mice. ‡ indicates a significant difference between control and test conditions within the same group of mice. Error bars are SEM.

Movement path vs. time plots show the exploratory paths overlying the video image of the arena with time plotted along the z-axis (Figure 2A-C, J-L). Visual comparison of the path vs. time plots shows that both *Ube3a *deficient mice had shorter exploratory paths and covered less area in their exploration than wild-type mice under both control (Figure 2A-C) and test conditions (Figure 2J-L). The quantitative comparison of path length under control and test conditions revealed that *Ube3a*^m-/p- ^and *Ube3a*^m-/p+ ^mice had significantly shorter exploratory paths than their wild-type litter mates, under both control and test conditions (Figure 3A) (*F [5,138] *= 33.29, *p *≤ 0.001, Tukey post hoc (TPH) results for pairwise comparison to wild type litter mates under control conditions: *Ube3a*^m-/p-^, *p *≤ 0.001, *mdiff *= -1690.23, *serr = *242.46 and *Ube3a*^m-/p+^, *p *≤ 0.001, *mdiff *= -1455.57, *serr *= 183.22; TPH results for pairwise comparison to wild type litter mates under test conditions: *Ube3a*^m-/p-^*, p *≤ 0.001, *mdiff *= -1223.17, *serr *= 242.46; *Ube3a*^m-/p+^, *p *≤ 0.001, *mdiff *= -1061.58, *serr *= 183.22). Similarly, quantitative comparison of the area covered by the paths (Figure 3B) showed that wild-type mice covered a significantly larger proportion of the arena than *Ube3a *deficient mice (*F [5,134] *= 20.89, *p *≤ 0.005, TPH. results for pairwise comparison to wild type litter mates under control conditions: *Ube3a*^m-/p-^, *p *≤ 0.002, *mdiff *= -8.16, *serr *= 2.07; *Ube3a*^m-/p+^, *p *≤ 0.005, *mdiff *= -5.81, *serr *= 1.6; TPH results for pairwise comparison to wild type litter mates under test conditions: *Ube3a*^m-/p+^, *p *≤ 0.001, *mdiff *= -8.22, *serr *= 1.6; *Ube3a*^m-/p-^, *p *≤ 0.001, *mdiff *= -9.01, *serr *= 2.07). There were no significant differences in path length or area covered between *Ube3a*^m-/p- ^and *Ube3a*^m-/p+ ^mice under either condition.

Color maps for duration of stay (Figure 2D-F, M-O) show sites were mice lingered for more than 60 seconds as red and purple spots (two examples marked by arrows in Figure 2D). Both *Ube3a*^m-/p- ^and *Ube3a*^m-/p+ ^mice (Figure 2D-F) lingered frequently for 60 seconds or more at various sites distributed across most of the two side chambers with a preference of lingering close to a wall (Figure 2D,E). Mice rarely lingered in the middle chamber.

By comparison, the wild-type mouse had overall fewer places where it lingered for more than 60 seconds and lingering sites were concentrated in front of the two empty cages (Figure 2F). A quantitative comparison of lingering behavior as the proportion of time the mice spent moving during the 10 min trials revealed that wild-type mice spent significantly more time moving than did *Ube3a *deficient mice (*F [5,138] *= 30.99; *p *≤ 0.001; TPH results for pairwise comparison to wild type litter mates under control conditions: *Ube3a*^m-/p-^, *p *≤ 0.001, *mdiff *= -13.77 *serr *= 2.33; *Ube3a*^m-/p+^, *p *≤ 0.001, *mdiff *= -11.62, *serr *= 1.76, TPH results for pairwise comparison to wild type litter mates under test conditions: *Ube3a*^m-/p-^, *p *≤ 0.001, *mdiff *= -10.02, *serr *= 2.33; *Ube3a*^m-/p+^, *p *≤ 0.001, *mdiff *= -9.33, *serr *= 1.76). Comparison of the color maps for speed (Figure 2 G-I, P-R) show that *Ube3a *deficient mice generally moved at a slower speed than their wild-type littermates. Quantitative comparison revealed significantly lower movement speeds in both *Ube3a *deficient genotypes compared to wild-type littermates (*F [5,138] *= 32.52, *p *≤ 0.001; TPH results for pairwise comparison to wild type litter mates under control conditions: *Ube3a*^m-/p-^, *p *≤ 0.001, *mdiff *= -2.83, *serr *= 0.41; *Ube3a*^m-/p+^, *p *≤ 0.001, *mdiff *= -2.47, *serr *= 0.31; TPH results for pairwise comparison to wild type litter mates under control conditions: *Ube3a*^m-/p-^, *p *≤ 0.001, *mdiff *= -2.07; *serr *= 0.41; *Ube3a*^m-/p+^, *p *≤ 0.001, *mdiff *= -1.81, *serr = *0.31) (Figure 3D). This difference was independent of social context, i.e. of the presence or absence of a stranger mouse. There was no significant difference in movement speed between *Ube3a*^m-/p- ^and *Ube3a*^m-/p+ ^mice under either condition.

During the test trials (with a stranger mouse present) mice from all three genotypes spent extended amounts of time in front of the cage with the stranger mouse (cage marked "S" in Figure 2M-R) signified by the accumulation of lingering sites (red/purple dots). This was interpreted as social interest in the stranger mouse and was quantified as the total amount of time the mice spent inside the area in front of the cage. We also measured the number of visits or entries into the area in front of the stranger's cage. A detailed description of results for the behaviors directly in front of cages is given below.

We consistently observed that mice of all three genotypes moved slower, had shorter track lengths and spent less time moving during the second trial, i.e. when the stranger mouse was present in one of the cages. The decreases in these variables were comparable in magnitude between genotypes but significant differences for each variable were only seen in the wild-type mice (Figure 3) (TPH results for pairwise comparison between trials: Track length: *p *≤ 0.001, *mdiff *= -907.9, *serr *= 165.88; Velocity: *p *≤ 0.001, *mdiff *= -1.51, *serr *= 0.28; Activity: *p *≤ 0.001, *mdiff *= -10.03, *serr *= 1.59). We wanted to know whether this decrease in exploratory activity during the test trial was related to the presence of the stranger mouse or due to reduced drive to explore a now familiar arena. Control experiments were conducted in which the mice explored the arena twice without a stranger mouse present. All other procedures were kept identical to the social tests. These control experiments were conducted only with *Ube3a*^m-/p+ ^(*n *= 5) and wild-type (*n *= 5) mice, as we had not found any differences between *Ube3a*^m-/p- ^and *Ube3a*^m-/p+ ^in exploratory activity. During the second trial of the control experiments movement speed, track length and time spent moving decreased for both the *Ube3a*^m-/p+ ^and wild-type mice. The observed decreases were comparable in magnitude to those observed in the social experiments. Decreases in time spent moving reached significance for both *Ube3a*^m-/p+ ^and wild-type mice (*F [3,16] *= 27.92, *p *≤ 0.001; TPH results for pairwise comparison between trials: *Ube3a*^m-/p+^, *p *≤ 0.001, *mdiff *= -11.1, *serr *= 2.22, Ube3a^m+/p+^, *p *≤ 0.001, *mdiff *= -11.98, *serr *= 2.22). Thus, the observed decreases in exploratory activity in the second trial of the social test were not dependent on the presence of a stranger mouse.

### Behavior in front of cages

**Figure 4:**
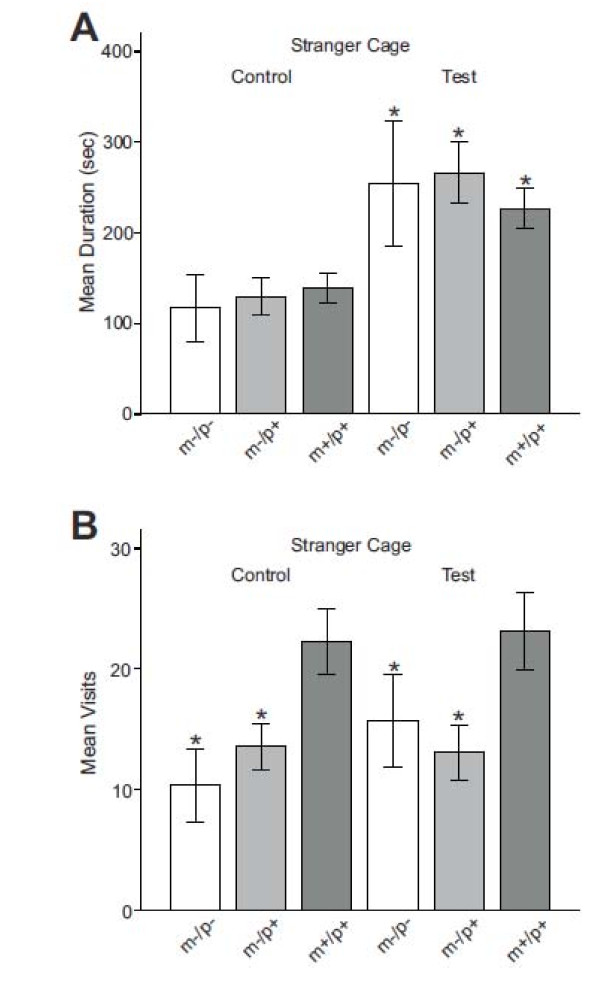
**Analysis of behavior in the area directly in front of the cages with and without the stranger mouse**. Each bar graph shows results from control trials on the left and from test trials (with stranger present) on the right. Bar colors correspond to genotypes (*Ube3a*^m-/p- ^= white bar, *Ube3a*^m-/p+ ^= light gray, wild-type = dark gray). (A) Time spent in front of the cage when the stranger was present (test trial) compared to the same cage when it was empty (control trial). Asterisks indicate significant differences between control and test conditions. (B) Mean number of visits to the stranger's cage during control and test conditions. Asterisks indicate a significant difference between the *Ube3a *deficient and the wild-type mice. Error bars are SEM.

Social interactions with the stranger mouse were quantified as the amount of time the test mice spent in a small area directly in front of the stranger's cage (shaded areas in Figure 1B). Comparison of the total time mice spent directly in front of either cage (duration) revealed that mice of all genotypes spent significantly more time in front of the stranger's cage than in front of the empty cage (Figure 4A) (*F [11,272] *= 18.62, *p *≤ 0.001; TPH results for pairwise comparison between cages during presence of a stranger mouse (test conditions): *Ube3a*^m-/p-^, *p *≤ 0.001, *mdiff *= 137.7, *serr *= 28.8; *Ube3a*^m-/p+^, *p *≤ 0.001, *mdiff *= 136.67, *serr *= 19.5; Ube3a^m+/p+^, *p *≤ 0.001, *mdiff *= 87.54, *serr *= 15.92). There were no significant differences between wild type and *Ube3a *deficient mice for the amount of time spent in front of the stranger's cage. Under control conditions, mice of all genotypes spent the same amount of time in front of both cages, showing that there was no preexisting preference for either cage.

Analysis of the number of visits, i.e. the number of times mice walked into the areas in front of the cages, revealed significant differences between groups (Figure 4B). Under control conditions both *Ube3a*^m-/p- ^and *Ube3a*^m-/p+^, had significantly fewer visits to either of the two cages than wild-type mice (*F [11,272] *= 13.96, *p *≤ 0.001; TPH results for pairwise comparison with wild-type litter mates for the cage that was empty during control and test trials: *Ube3a*^m-/p-^, *p *≤ 0.015, *mdiff *= -8.57, *serr *= 2.33; *Ube3a*^m-/p+^, *p *≤ 0.004, *mdiff *= -7.15, *serr *= 1.79. TPH results for pairwise comparison with wild-type litter mates for the cage that was empty during control but housed the stranger mouse during test trials: *Ube3a*^m-/p-^, *p *≤ 0.001, *mdiff *= -11.94, *serr *= 2.33; *Ube3a*^m-/p+^, *p *≤ 0.015, *mdiff *= -8.72, *serr *= 1.79). Also with the stranger present, *Ube3a *deficient mice still visited the stranger's cage significantly less often than the wild-type mice (*F [11,272] *= 13.96; TPH results for pairwise comparison with wild-type litter mates: *Ube3a*^m-/p-^, *p *≤ 0.041 *mdiff *= -7.84, *serr *= 2.33, *Ube3a*^m-/p+^, *p *≤ 0.001, *mdiff *= -8.72, *serr *= 1.79).

The analysis of the paths within the areas in front of the cages revealed similar differences between *Ube3a *deficient and wild type mice in movement speed, exploratory path lengths and time spent moving found for the entire path. *Ube3a *deficient mice spent significantly less time moving in the areas in front of the cages than their wild-type litter mates under control and test conditions (*F [11,272] *= 20.89, *p *≤ 0.001; TPH results for pairwise comparison with wild-type litter mates during control conditions: *Ube3a*^m-/p-^, *p *≤ 0.001, *mdiff *= -10.72, *serr *= 2.02; *Ube3a*^m-/p+^, *p *≤ 0.001, *mdiff *= -9.06, *serr *= 1.54. TPH results for pairwise comparison with wild-type littermates during test conditions: *Ube3a*^m-/p-^, *p *≤ 0.001, *mdiff *= -8.73, *serr *= 2.02; *Ube3a*^m-/p+^, *p *≤ 0.001, *mdiff *= -7.96, *serr *= 1.54). There was no significant difference between *Ube3a*^m-/p- ^and *Ube3a*^m-/p+ ^mice in any of the variables. As before, these differences were in not affected by the presence of the stranger mouse.

We also analyzed freezing behavior in the whole field and in front of the cages. There was no significant difference in the number of freezing events between any of the genotypes or between experimental conditions.

## Discussion

We tested mice with either maternal (*Ube3a*^m-/p+^) or complete (*Ube3a*^m-/p-^) loss of *Ube3a *expression for their social interest in a stranger mouse as well as for their levels of activity and patterns of exploratory behavior. The main findings of our study are that social seeking behavior and hyperactivity, both of which are characteristic behavioral phenotypes of human Angelman syndrome patients [1,2,15,16], were not observed in either form of *Ube3a *deficient mice. *Ube3a *deficient mice showed the same interest in spending time with a stranger mouse as did their wild-type littermates, indicating normal social behavior. The exploratory behavior of *Ube3a *deficient mice revealed reduced activity compared to their wild-type littermates. Thus, in contrast to Angelman syndrome, were hyperactivity is consistently described as a behavioral phenotype, complete or maternal loss of *Ube3a *expression in mice results in hypoactivity. Loss of *Ube3a *expression also causes mild cerebellar ataxia in both humans and mice [1,2,10,13]. Considering possible effects of ataxia on the measurement of activity it is important to note that the activity was determined as the amount of time the mice spent moving faster than a very low minimum speed (0.10 cm/sec). Analysis of exploratory paths showed that *Ube3a *deficient mice were clearly able to move much faster than the threshold-speed for activity registration. Thus, ataxia is unlikely to be the cause of hypoactivity in mice.

Interestingly, the numbers of visits to the areas in front of the cage with the stranger mouse were not different in any genotype for control (no mouse present) and test conditions (stranger mouse present). This suggests that the presence of the stranger mouse did not cause any of the mice to change their exploratory path to increase the number of encounters, but instead that visits to the cages were rather chance events during exploration. The extended stay in front of the stranger's cage, however, was clearly related to the presence of the stranger. We did see, however, that *Ube3a *deficient mice visited the cages significantly less often than did wild type mice. We suggest that this difference is not related to social context but rather due to the difference in overall exploratory activity. The difference in the number of visits to the areas in front of the cages amounted to 37.7% fewer visits of *Ube3a *deficient compared to wild-type mice. This reduction is of similar magnitude as the 37.3% difference in the amount of time spent moving (with *Ube3a *deficient mice moving less).

All mice showed a general reduction in various measures of activity (speed, track length, time spent moving) during the second or test trial. However, those reductions in activity were also observed in control experiments where no stranger mouse was present during the second trial. Thus, the reduced activity was independent of the stranger's presence and likely the result of reduced drive to explore the then familiar arena during the second trial.

In order to determine the value of the *Ube3a *deficient mouse as a model for Angelman syndrome it will be important to understand whether the phenotypic differences between patients and *Ube3a *deficient mice reported here are based on species specific effects of loss of *Ube3a *expression, on differences in the extent of the genetic defects or on a combination of both. It is important to point out, that the majority of Angelman patients have contiguous gene deletions, i.e. have lost expression of more than one gene. There are several molecular classes of Angelman syndrome including 15q11-q13 deletion, uniparental disomy, imprinting defects and *UBE3A *gene mutation. The most common of these is a deletion of maternal origin on chromosome 15q11-q13, which accounts for about 70% of AS cases [17]. Patients with the deletion phenotype tend to have a more severe phenotype, for example severe microcephaly, more severely impaired communication and more severe seizures than patients with other molecular classes of *UBE3A *deficiency [17]. These deletions can be further categorized into class I and class II deletions. Patients with the larger class I deletions that encompass the locus for the FRMP interacting protein CYFIP1 appear to have an increased risk for autism as compared to patients with class II deletions [8]. It should also be noted that individuals with Prader-Willi Syndrome (PWS) as a result of maternal uniparental disomy (i.e. two maternal copies of 15 and thus no paternal specific expression at the *SNRPN *locus) have also been reported as having more autism like features than PWS deletion individuals [18]. These phenotypic differences among deletion, mutation and even uniparental disomy individuals may help to explain some of the conflicting literature in regards to social behavior observed in Angelman syndrome. Similarly, the fact the *Ube3a *deficient mice lack expression of only one gene might be at least partially responsible for the phenotypic differences between Angelman patients and *Ube3a *deficient mice reported here. A recently published study used a new mouse model with a large maternal deletion from *Ube3a *to *Gabrb3*. This mouse model reflects the contiguous human gene deletions more closely and a comparative study between both genetic models would be informative about the role of specific role of Ube3a in hyperactivity, social and other behaviors.

All Angelman syndrome patients have significant developmental delay in cognitive and motor functions [3,19]. Weather the behavioral patterns observed here in adult mice changes with age and weather those changes parallel development in humans is thus another important question to address in follow-up studies.

## Conclusions

Although the mouse model of *Ube3a *deficiency used here shares physiological and phenotypic features with Angelman patients, we show that social seeking behavior and hyperactivity are not reproduced. The majority of AS patients have a maternally derived deletion, which encompasses several genes including *UBE3A*. Individuals with maternally inherited mutations affecting only *UBE3A *tend to have a milder phenotype than the individuals with deletions [6]. The mice tested here are more similar to the individuals with point mutations since they are lacking only the *Ube3a *protein and do not have a contiguous gene deletion [11]. It remains to be shown whether the phenotypical differences between *Ube3a *deficient mice and Angelman patients are based on species-specific differences, differences in genetic conditions or a combination of both.

## Methods

The construction and breeding of the *Ube3a *deficient mice used for these studies have been previously described [11] and these animals have previously been used for behavioral studies by our group [13]. The mice used for this study were 98% congenic to C57BL/6J according to SNP genotyping performed at Jackson Laboratory (Bar Harbor, Maine). All mice used in this study were raised and all experiments performed in accordance with procedural guidelines approved by the University of Tennessee Health Science Center Animal Care and Use Committee. Principles of laboratory animal care (NIH publication No. 86-23, rev. 1996) were followed.

All behavioral tests were performed with adult male mice 8 - 12 weeks of age that were either homozygous for the Ube3a null allele (*Ube3a*^*m*^^-/p-^, *n *= 11) or inherited the null allele through the maternal germline (*Ube3a*^*m*^^-/p+^, *n *= 25, except for measurements of area covered where *n *= 23). Wild-type littermates (Ube3a^m+/p+^, *n *= 36) of these animals were used as controls. Mice were housed in standard cages with ad libitum food and water access with 12-hour light/dark cycles. Mice used as strangers in the social test were wild-type C57BL/6 mice that were unrelated to the test animals and had not been previously in contact with test animals.

### Social Behavior Test

The social behavior test was designed based on the test developed by Moy et al. [14,20] to quantitatively evaluate the interaction of the test mice with a stranger mouse and at the same time evaluate exploratory behavior. Tests were performed in a rectangular Plexiglas arena (63 cm L × 42 cm W × 22 cm H), which was subdivided in to three rectangular chambers of equal size (21 cm L × 42 cm W). An outline of the arena is shown in Figure 1. The walls separating the chambers had centered openings (dashed lines in Figure. 1) through which the mice could freely move between chambers. The two side chambers each contained a small cage (21 cm L × 10 cm W × 22 cm H) built from 1.5 cm^2 ^grid cage wire-mesh. The mouse's movement path was continuously tracked with an automatic video tracking system (BIObserve GmbH, Bonn, Germany). The digitized track coordinates as well as various derived variables such as speed, duration, activity and freezing, were calculated and stored on hard disk for statistical comparison. Movements of the stranger mice in the small cages were not tracked.

The social behavioral test consisted of two consecutive 10-minute trials. Each trial started with the test mouse being placed in the center chamber. For consistency, test mice were set down in the center chamber facing the same general direction (the long outer wall of the arena with the two small cages). During the first trial the two small cages were empty. The test mice explored the arena freely and their exploratory paths were tracked and digitized. Mice were then briefly removed from the arena and placed in a neutral "waiting" cage with fresh bedding while a stranger mouse was placed in one of the two small cages. Cage choice was varied randomly between trials. Then, the test mice were again placed into the center chamber of the arena. Social interactions with the stranger mouse were evaluated based on the amount of time the test mice spent directly in front of the cage with the stranger (see below for details of trajectory analysis).

After completion of each test, the bedding was removed, the arena was wiped down with water and new bedding was put in place. The small metal cages were hosed cleaned with hot water using high pressure and dried before placing them back into the arena. The experimenter was present and observed the mouse's movements for the entire duration of the experiments. The test could not be run unsupervised because occasionally the test mice would attempt to climb onto the cages in the side chambers. If they reached the top of the cage the experimenter used a plastic rod to gently direct them back towards the arena.

### Quantitative analysis of exploratory path

In all tests, mouse movements were analyzed using the automatic video tracking and analysis software Viewer II (BIObserve GmbH, Bonn, Germany). A number of quantitative measures (listed below) were derived from the movement path analysis statistical comparison. These values were calculated for the whole arena (shaded area in Figure 1a) and for each of the two small areas immediately in front of the small cages (shaded rectangular areas in Figure 1b).

Exploratory behavioral variables were defined as follows:

*Time spent moving*: The percentage of time the mouse was in motion during the 10-minute trials. The speed threshold for motion vs. rest was 0.1 cm/sec

*Speed*: Average movement speed over the entire 10-minute trial duration (in cm/sec).

*Track length*: Total length of the exploratory path (in cm).

*Area covered*: For this analysis the arena was subdivided into small 1.2 cm2 grids. The total area covered by the exploratory path was calculated as the percentage of grid elements crossed during exploration.

*Duration*: The time spent at any given site along the path (in sec).

*Freezing*: The number of events where the animal would be immobile for more than 2 sec (speed < 0.5 cm/s).

The following analyses were specific for the areas in from of the small cages:

*Number of visits: *The number of times the mouse walked into the area in front of the empty or the "stranger's" cage.

*Duration*: The total amount of time the mice spent inside the areas in front of the empty or the "stranger's" cage.

The results were statistically evaluated using analysis of variance (ANOVA) followed by Tukey Post Hoc test (SPSS v. 17, SPSS Inc., Chicago, IL). F statistics results are reported with between- and within- groups degrees of freedom. All *p *values are reported as inequalities (e.g. *p *≤ 0.01). We used a 95% confidence interval for significance (i.e. *p *< 0.05). We report Tukey Post Hoc (TPH) test results for all significant pair-wise comparisons including the *p *values and the mean (*mdiff*) and standard (*serr*) of the difference to the reference group or test condition.

The exploratory paths as well as speed and duration measurements were visualized in two- and three-dimensional maps (Figure 2). In Figures 2A,B,C the exploratory paths are plotted as space vs. time representations. Travel speed and duration are represented in color code in Figures 2D,E,F and 2G,H,I respectively. High speed and duration values are shown in "hot" colors (e.g. red, yellow) and low values in "cold" colors (e.g. blue, green).

## Authors' contributions

MA carried out the behavioral assays, performed the statistical analysis, contributed to the design of the study and the writing of the manuscript. AS participated in the behavioral assays and contributed to the design of the study. LTR provided the mice and contributed to the preparation of the manuscript. DHH conceived of and coordinated the study and wrote the manuscript. All authors read and approved the final manuscript.
